# Lymphatic malformations: formerly known as lymphangiomas. A rare case of two breast masses in different locations mimicking breast carcinomas

**DOI:** 10.31744/einstein_journal/2026RC2042

**Published:** 2026-04-30

**Authors:** Laura Mulazzani Minuzzi Macedo, Fernando Camargo Fernandes, Ana Carolina Dourado Mattos Raful, Heni Debs Skaf, Marcela Caetano Vilela Lauar, Erica Elisângela Françolin Federicci

**Affiliations:** 1 Hospital Israelita Albert Einstein São Paulo SP Brazil Hospital Israelita Albert Einstein, São Paulo, SP, Brazil.; 2 Hospital Israelita Albert Einstein Faculdade Israelita de Ciências da Saúde Albert Einstein São Paulo SP Brazil Faculdade Israelita de Ciências da Saúde Albert Einstein, Hospital Israelita Albert Einstein, São Paulo, SP, Brazil.; 3 Hospital Israelita Albert Einstein Hospital Municipal Gilson de Cássia Marques de Carvalho São Paulo SP Brazil Hospital Municipal Gilson de Cássia Marques de Carvalho; Hospital Israelita Albert Einstein, São Paulo, SP, Brazil.

**Keywords:** Lymphatic abnormalities, Lymphatic system, Breast diseases, Magnetic resonance spectroscopy, Ultrasonography

## Abstract

Lymphatic malformations are benign disorders of the lymphatic system and represent a rare diagnosis in adulthood, particularly when involving the breast. This report describes the case of a 64-year-old patient referred to our service with two suspicious masses located in different areas of right breast. Imaging studies reinforced the suspicion of malignancy, and further investigation was conducted with an ultrasound-guided core needle biopsy, which confirmed the diagnosis of lymphatic malformation. As illustrated in this report, although lymphatic malformation may present with imaging findings, histopathological evaluation is often required to exclude malignancy before complete surgical excision. Therefore, lymphatic malformation should be considered as a potential differential diagnosis in the investigation of breast masses suspicious for breast cancer.

## INTRODUCTION

In 2018, the International Society for the Study of Vascular Anomalies (ISSVA) published an international consensus on the nomenclature and classification of vascular anomalies, including lymphatic malformations. The significant change was the primary division of vascular anomalies into tumors and malformations. Vascular tumors include pathologies characterized by abnormal proliferation of endothelial cells with neoplastic behavior, whereas vascular malformations comprise structural vascular abnormalities with quiescent endothelial cell proliferation.^(1,2)^ Vascular malformations are subdivided into "fast-flow", "slow-flow", and "developmental anomalies of named vessels". Lymphatic disorders are classified within the "slow-flow"category.^(2,3)^ Accordingly, previously used and imprecise terms such as "lymphangiomas", "lymphatic cyst" and "cystic hygroma" were standardized and replaced by the term "lymphatic malformations".^(4)^

Considering the context described above, lymphatic malformations are slow-flow benign vascular anomalies that arise from errors in vascular development.^(5)^ This condition is rarely diagnosed in adulthood and is primarily identified during childhood, with 50% of cases being detected at birth and 90% of cases occurring by two years of age.^(6)^ These lesions may affect any part of the body but occur most commonly in the head and neck region (75%) or axillae (20%).^(7)^ The breast is an infrequent site of origin for lymphatic malformations, and only a limited number of cases have been reported in the literature.^(7,8)^ As described in reports, when the breast is involved, the condition most often presents as a single mass^(7)^ located typically in the retroareolar region or in the upper outer quadrant, which is likely related to the anatomical pattern of the breast lymphatic drainage.^(8)^

The pairing of imaging exams with typical findings helps in the diagnosis of this condition. However, there are cases in which ruling out malignancy remains a challenge. This report describes an unusual case of two lymphatic malformations in different locations in the same breast (upper and lower outer quadrants), with suspicious findings on both magnetic resonance imaging (MRI) and ultrasound, resembling breast carcinoma.

## CASE REPORT

A 64-year-old female patient with a history of hypertension and diabetes, a former smoker, not using hormone replacement therapy, and with a family history of breast cancer (mother), was referred to our breast imaging department after an initial external evaluation of two palpable masses that had been progressively growing for approximately 2 years, without other systemic complaints or symptoms. Prior to referral, she had undergone external ultrasound and mammography, both of which recommended further investigation with biopsy. However, the procedure was postponed and not performed until evaluation at our service due to an episode of acute myocardial infarction.

**Figure 1:**
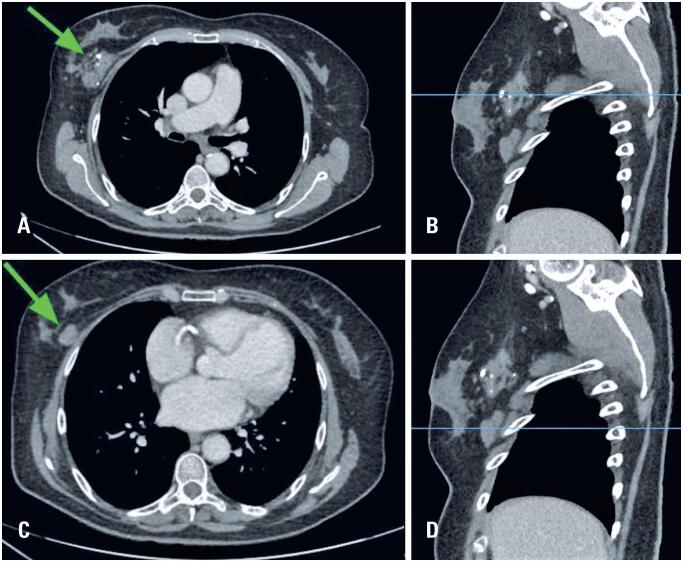
Chest CT showing two irregular hyperdense masses (green arrows) with hyperattenuating foci in the right breast: in the upper outer quadrant (A - axial view, B - sagittal view) and in the lower outer quadrant (C - axial view; D - sagittal view), in the right breast. The blue lines in figures B and D indicate the correlation between the findings observed in the sagittal planes and those in the axial planes

In our department, clinical assessment revealed a palpable mass larger than 6 centimeters located in the upper outer quadrant of the right breast, as well as a smaller mass in the lower outer quadrant, both with imprecise borders. There was no skin involvement, including erythema, cellulitis, skin thickening, nipple retraction, or breast tenderness. Due to complaint of chest pain, computed tomography (CT) scan of the chest was requested which demonstrated the presence of masses in the outer quadrants of the right breast parenchyma, with interspersed calcified foci (Figure 1).

**Figure 2:**
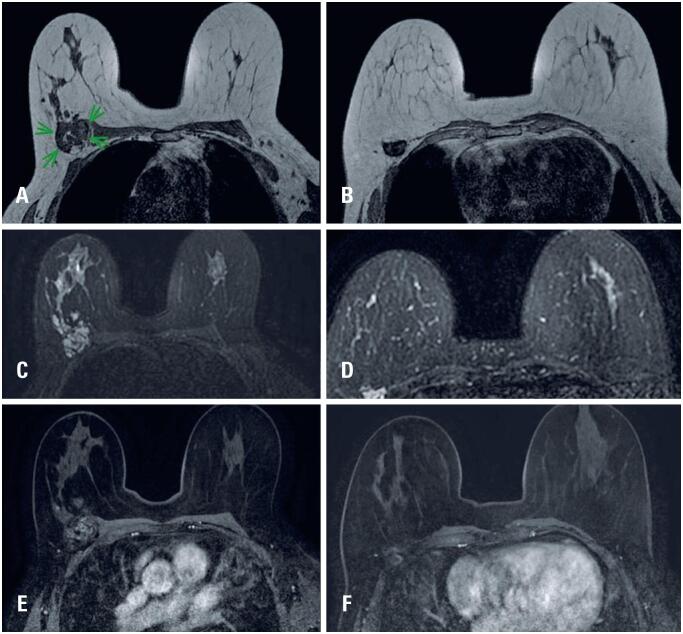
T1-weighted breast MRI showing two irregular masses in the posterior third of the upper outer (A) and lower outer (B) quadrants of the right breast. T2-weighted breast MRI showing both lesions (C, - upper outer quadrant, D, -lower outer quadrant) with high signal intensity. Post-contrast subtraction images demonstrate heterogeneous and persistent enhancement of the lesions (E, - upper outer quadrant; F, - lower outer quadrant), without evident enhancement the pectoralis major muscle

For further evaluation, a breast MRI scan was performed, which revealed two masses with irregular margins and morphology in the posterior third of the upper and lower outer quadrants of the right breast, as shown on T1-weighted sequences (Figure 2A and 2B).

Both lesions showed high signal on T2-weighted sequences, measuring 6.5x4.2x3.3cm (upper outer quadrant) and 2.7x2.1x1.9cm (lower outer quadrant), adjacent to the pectoral muscles, but without signs of muscle invasion (Figure 2C and 2D). Dynamic contrast enhanced sequences revealed heterogeneous enhancement with persistent kinetics, with no evident enhancement of the pectoralis major muscle (Figure 2E and 2F). Additional examinations, including abdominal computed tomography and bone scintigraphy, were performed and showed no noteworthy findings relevant to the investigation.

**Figure 3:**
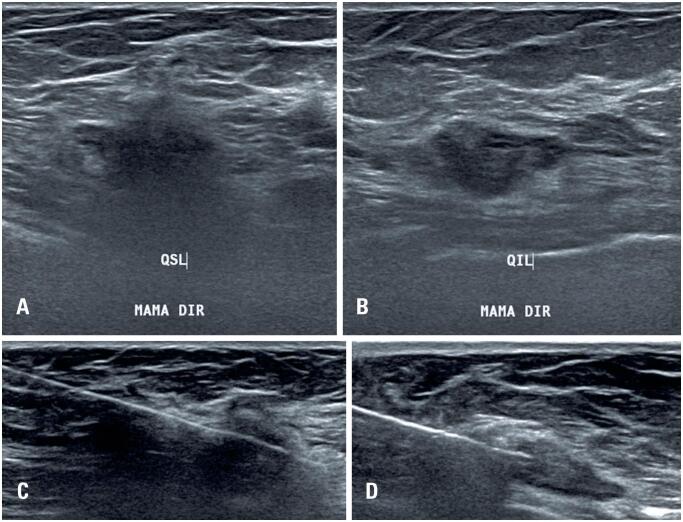
Ultrasound of the right breast showing two hypoechoic, irregular masses with spiculated margins, malignancy, located in the upper outer quadrant (A) and lower outer quadrant (B) Ultrasound-guided core needle biopsy was subsequently performed (C - upper outer quadrant, D - lower outer quadrant)

Ultrasound revealed hypoechoic, irregular masses with spiculated margins, suspicious for malignancy. Ultrasound-guided core needle biopsy of both lesions was subsequently performed (Figure 3).

**Figure 4:**
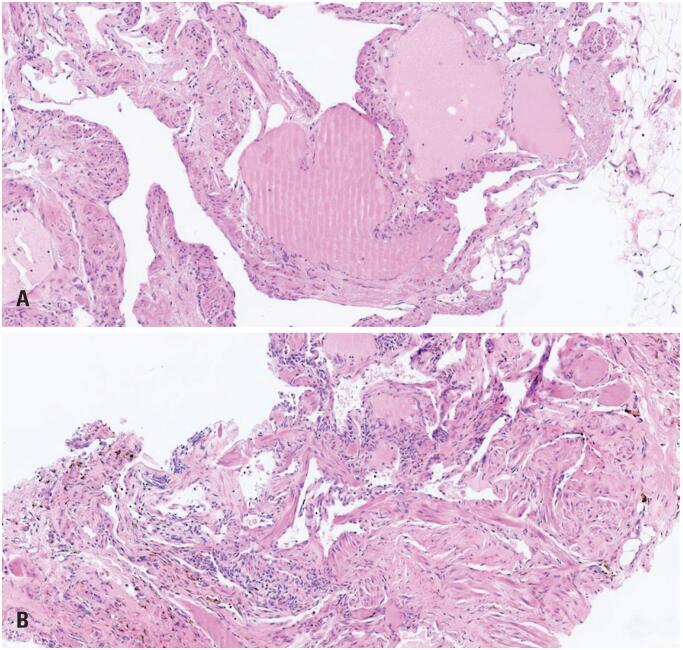
Histopathological analysis revealed ectatic lymphatic vessels lined by endothelial without atypia and stroma with fibroelastic pattern and aggregation of hemosiderophages, compatible with isolated lymphatic malformation

Histopathological analysis (Figure 4) revealed proliferation of ectatic lymphatic vessels lined by endothelial cells, without atypia, and stroma with a fibroelastic pattern, along with aggregation of hemosiderophages, findings compatible with an isolated lymphatic malformation. The immunohistochemical study demonstrated positive expression for the EP111 and AE1/AE3 antibodies.

Due to the personal history of heart failure, with a planned heart transplant and a worsening ejection fraction after an acute myocardial infarction six months prior to the diagnosis of lymphatic malformation, radiological follow-up of the lesions was chosen. On a follow-up ultrasound performed three months after the diagnosis, the lesions remained stable, with no evidence of growth. The patient was referred for surgical risk assessment but was considered unfit for surgery due to the high anesthetic-surgical risk associated with mastectomy for lesion removal.

Subsequently, attempts to contact the patient were unsuccessful. A family member later reported that the patient had died from complications of heart disease four years after being lost to follow-up at our service.

### Ethical aspects

This study was approved by the Research Ethics Committee of *Hospital Israelita Albert Einstein*, in accordance with the principles outlined in CNS Resolution No. 466/2012 and registered under Certificate of Ethical CAAE: 88573425.3.0000.0071; # 7.563.736. The requirement for informed consent was waived by the committee due to the patient's death.

## DISCUSSION

Lymphatic malformations are slow-flow vascular malformations that arise due to errors in vascular development^(5)^ or secondary to trauma, infections, chronic inflammatory processes, or obstructions.^(9)^ Their physiopathology is related to abnormalities in the development and proliferation of the lymphatic system, as well as in its connection to the veno-lymphatic system. According to the current ISSVA classification, lymphatic malformations are divided into three categories: isolated, complex, and lymphedema. Isolated lymphatic malformations, as presented in this case, refer to localized lesions, usually single, composed of anomalous lymphatic channels or cysts, without systemic or multifocal involvement. These lesions may be macrocystic, microcystic, or mixed. Complex lymphatic malformations encompass a spectrum of anomalies involving multiple territories and may be associated with specific syndromes. Finally, primary lymphedema is defined as the chronic accumulation of interstitial fluid resulting from the lymphatic system's inability to adequately drain lymph.^(4,10)^

Regarding the imaging findings of isolated lymphatic malformations, mammographic findings are nonspecific,^(8)^ typically presenting as hyperdense masses with variable margins and morphology. Ultrasound is helpful in evaluating the cystic components of the lesion: macrocystic lesions (>2cm) tend to be multiloculated, with thin septa, and may appear as mixed solid and cystic; Microcystic lesions (<2cm) present as multiple small cysts, often infiltrative, however, the cysts may occasionally be too small to distinguish the lesions to appear nonspecific. Mixed lesions include combined macrocystic and microcystic areas.^(10)^

Magnetic resonance imaging is the modality of choice for the diagnosis and assessment of disease extent, since it accurately delineates the radiological findings and the involvement of adjacent structures. On MRI, the most common pattern is a multiloculated cystic lesion, usually with an irregular shape, well-defined margins, and infiltrative growth. The cystic content typically exhibits hypointensity on T1-weighted images and hyperintensity on T2-weighted images, reflecting serous or lymphatic fluid. The walls and internal septa are usually thin and may show enhancement after contrast administration, whereas the cystic content does not enhance after contrast. ^(11)^ Finally, it should be emphasized that lymphatic malformations are rare and present no pathognomonic imaging findings.

As described in this report, lymphatic malformations may present imaging features suspicious for malignancy, and can mimic breast carcinoma, since both conditions may radiologically appear as voluminous, multiloculated lesions with septa, contrast enhancement, and infiltrative behavior.^(12)^ Therefore, as performed in this case, biopsy is essential to establish the correct diagnosis.

Although there are different therapeutic options, such as incision and drainage, sclerotherapy, steroids, radiotherapy, or carbon dioxide laser, complete surgical excision remains the most effective treatment modality,^(7)^ as it reduces the risk of recurrence and complications.

## CONCLUSION

This report describes an unusual case of isolated lymphatic malformations that presented as lesions suspicious for malignancy on breast imaging. Although lymphatic malformations may present with typical imaging findings, biopsy may be necessary to exclude breast cancer before complete surgical excision. Thus, this condition should be considered as a possible differential diagnosis in the evaluation of breast masses suspicious for breast cancer.

## Data Availability

The data supporting the findings of this study are contained within the manuscript and will be publicly available upon publication of the article.
